# Whole exome sequencing reveals putatively novel associations in retinopathies and drusen formation

**DOI:** 10.1038/s41431-021-00872-3

**Published:** 2021-03-29

**Authors:** Lance P. Doucette, Nicole C. L. Noel, Yi Zhai, Manlong Xu, Oana Caluseriu, Stephanie C. Hoang, Alina J. Radziwon, Ian M. MacDonald

**Affiliations:** 1 Department of Ophthalmology & Visual Sciences, Faculty of Medicine & Dentistry, University of Alberta, Edmonton, AB Canada; 2 Department of Medical Genetics, Faculty of Medicine & Dentistry, University of Alberta, Edmonton, AB Canada

**Keywords:** Genetics, Next-generation sequencing

## Abstract

Inherited retinal dystrophies (IRDs) affect 1 in 3000 individuals worldwide and are genetically heterogeneous, with over 270 identified genes and loci; however, there are still many identified disorders with no current genetic etiology. Whole exome sequencing (WES) provides a hypothesis-free first examination of IRD patients in either a clinical or research setting to identify the genetic cause of disease. We present a study of IRD in ten families from Alberta, Canada, through the lens of novel gene discovery. We identify the genetic etiology of IRDs in three of the families to be variants in known disease-associated genes, previously missed by clinical investigations. In addition, we identify two potentially novel associations: *LRP1* in early-onset drusen formation and *UBE2U* in a multi-system condition presenting with retinoschisis, cataracts, learning disabilities, and developmental delay. We also describe interesting results in our unsolved cases to provide further information to other investigators of these blinding conditions.

## Introduction

Inherited retinal dystrophies (IRDs) affect ~1 in 3000 individuals and account for a large proportion of untreatable blinding disorders, however many conditions remain without a genetic explanation [1]. IRDs affect the light detecting cells of the retina, rod and/or cone photoreceptors, and may present as either non-syndromic or as a multisystem disorder (i.e. Bardet-Biedl syndrome). Advanced technologies such as next generation sequencing (NGS) have significantly improved our ability to both identify novel associations in family-based studies and provide genetic diagnoses in patients with IRDs. Currently, the proportion of IRD patients that receive a genetic diagnosis from a panel is variable (37–74%) [2]. Whole exome sequencing (WES) provides a hypothesis free first approach to identification of the genetic etiology of a condition, or as a research tool to identify novel genes and associations. The workflow for filtering and interpreting results of WES needs to be understood and appreciated, as not all investigations will result in the identification of previously documented variations in disease-associated genes. We report our experience with WES in Alberta, Canada using a cohort of ten families with heritable ocular disease selected for potential novel gene discovery. Through this study, we solved three families, which either confirmed or altered diagnoses, and found two potentially novel associations: variants in the low-density lipoprotein receptor related protein 1 (*LRP1*) gene in patients with macular drusen formation and in the *ubiquitin conjugating enzyme E2* (*UBE2U*) gene in a pedigree of retinal dystrophy with associated systemic defects. In addition, we described potential associations in our unsolved cases, which will assist further investigations into these conditions.

## Materials & methods

### Family selection

**Fig. 1:**
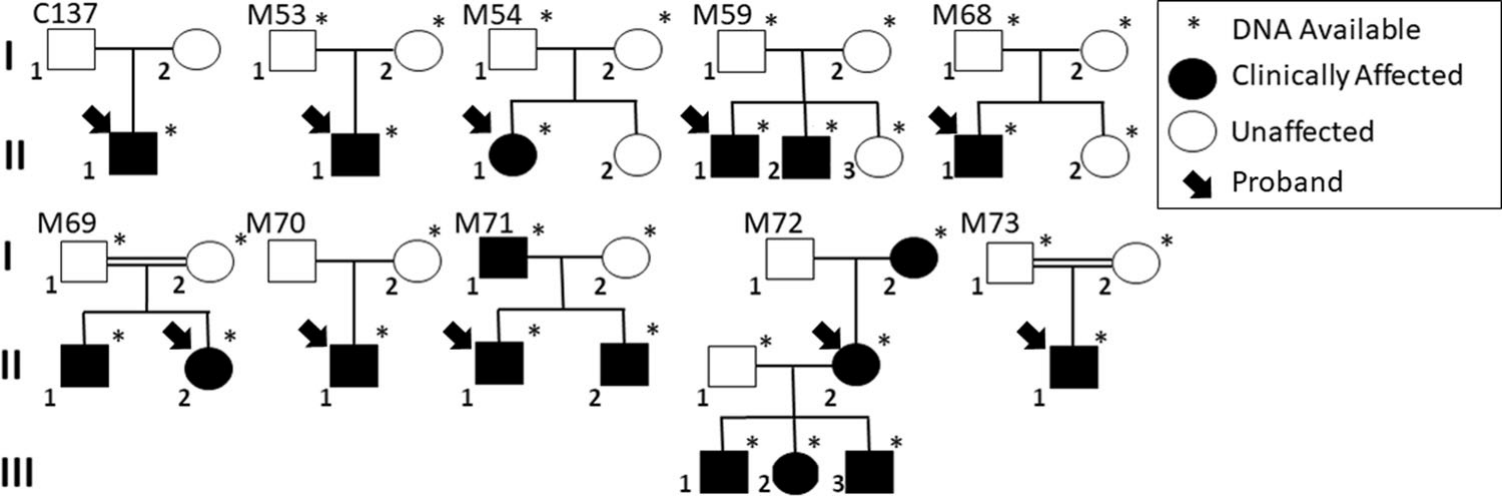
Pedigrees of selected families for WES study. Ten families were selected based on clinical diagnoses, availability of DNA/phenotypic information from family members, and previous negative clinical/research genetic testing. Shaded individuals indicate affected retinal conditions/syndromes further highlighted in Table 1.

Families and individuals were selected based on clinical diagnoses, availability of DNA/phenotypic information from family members, and previous negative clinical/research genetic testing. In total, ten families were selected for our WES study and signed consent obtained. Clinical data on affected family members are listed in Table 1; pedigrees are presented in Fig. 1. This study was approved by the University of Alberta Human Ethics Office (Pro00045377). All procedures abide by the Declaration of Helsinki. Whole exome sequencing five micrograms of whole genomic DNA isolated from either saliva or blood from family members were sent to the Beijing Genomics Institute (BGI, Beijing, China) or DNALink (South Korea) for WES. BGI used the Agilent B5 (50 M) exome capture kit and was run on the BGISEQ-500 (Avg coverage of 100x), DNALink used the SureSelect Exome V5 QXT and was run on Illumina Hiseq2500 (Avg Coverage of 96x). WES filters were set for appropriate mode of inheritance, moderate and high impact variants (missense, splice, and nonsense variants), and a minor allele frequency (MAF) adjusted for rarity of the condition (<0.01 for rare conditions, <0.02 for more common presentations). Filtered gene lists were compared with a list of known retinal disease genes (RetNet https://sph.uth.edu/retnet/, Accessed May 31, 2020). Families who were not solved using this methodology were moved to a novel discovery pipeline, segregation analysis where possible, and in silico analyses (SIFT/PROVEAN [3, 4], PolyPhen-2 [5], MutationTaster [6], SplicePort [7], Human Splicing Finder [8]) for any variants. All variants and phenotypic data listed in this paper were submitted to the Leiden Open Variation Database (LOVD; https://www.lovd.nl/, submitted and accessed January 22nd, 2021).

**Table 1:** Affected individuals from ten families selected for WES studies.

Family ID	Individual ID	MOI	Age of diagnosis	BCVA	Fundus findings and ocular history	OCT	ERG	Ocular diagnosis	Non-ocular findings	Previous panel testing
C137	I-1	AD	72	OD 20/40, OS CF	Choroideremia like fundus in both eyes, mild pallor optic nerve head	Outer retinal layers loss, presence of outer retinal tubulations	Rod-cone dysdrophy in ffERG	Choroideremia	–	CHM negative
M53	II-1	AR	12	OD 20/150, OS 20/150	Initially normal, scattered flecks in macula	Atrophy of the ellipsoid zone in the central macular, decreased central retinal thickness	Reduced photopic b-wave ffERG, central loss in mfERG	Macular dystrophy	–	–
M54	II-1	AR	42	OD 20/20, OS 20/20	Multiple large, confluent drusen in the central macula and nasal retina	Numerous confluent drusen in the macula	Not available	Macular drusen	–	–
M59	II-1	AR	13	OD 20/50, OS 20/50	Mild cellophane OD, mild vessel attenuation	Irregularity of the outer retina with extensive atrophy of the ellipsoid zone, deposits at the level of the Bruch’s membrane-RPE complex	Rod-cone dystrophy, cone flicker reduced, severe reduction in all scotopic responses	Nonsyndromic retinitis pigmentosa	–	RP2, RP3 negative
M59	II-2	AR	12	OD 20/40, OS 20/50	Mild pigment mottling in macular	Parafoveal loss of the ellipsoid zone	Rod-cone dystrophy; cone flicker reduced; 10 Hz dim flicker not recordable	Nonsyndromic retinitis pigmentosa	Mild learning disability, post-axial polydactyly	RP2, RP3 negative
M68	II-1	AR	15	OD 20/40, OS 20/40	No specific findings	Normal OCT scan	Normal cone function and a mild reduction in the pure rod response in ffERG, central loss in mfERG	Macular dystrophy	–	MD Panel (BluePrint Genetics)
M69	II-2	AR	29	OD 20/200,OS 20/200	Circumscribed central retinal atophy	Atrophy of outer retina. Bruch’s membrane is absent in the central fovea.	Normal ffERG in the right eye; reduced cone flash and flicker in the left eye	Macular dystrophy	–	MD Panel (BluePrint Genetics)
M70	II-1	AR	36	OD 20/40, OS 20/25	Macular drusen	Numerous hyporeflective elevations of the retinal pigment epithelium in the central macula consistent with drusen	Not available	Macular drusen	–	–
M71	II-1	AD	24	OD LP, OS CF	Normal axial length. Right eye had retinal detatchment at age 10. Blind and painful right eye resulted in an enucleation. Pathology report revealed disorganized anterior chamber. Aphakia in the left.	Extensive atrophy of outer retina and decreased in central retinal thickness in the left eye.	Not available	Retinal detachment, retinal dystrophy	–	–
M72	II-2	AD						Bilateral congenital cataracts, nystagmus,	Bilateral hearing loss, breast cancer (31 yoa)	Cataract Panel (BluePrint Genetics)
M72	III-1	AD	11	OD CF, OS 20/500	Bilateral small optic nerves, nystagmus	Splitting between the inner nuclear and the outer plexiform layer in left eye consistent with retinoschisis	Not available	Bilateral congenital cataracts, nystagmus, retinoschisis OS	Mild to moderate hearing loss, learning disability (ADHD), moderate intellecutual disability, delayed developmental milestones, 25th–50th %ile height, 10–25th %ile weight, 3rd %ile head circumference, bracycephaly, micrognathia, 1 cafe au lait spot	Cataract Panel (BluePrint Genetics)
M72	III-2	AD	7	OD 20/50, OS 20/300	Normal	Not done	Left exotropia	Bilateral congenital cataracts, amblyopia, exotropia	Mild to moderate unilateral (left) hearing loss, moderate intelletual disability, delayed motor development, brachycephaly, micrognathia, 50th %ile height, 50th %ile weight, 3rd %ile head circumference. 1 cafe au lait spot	Cataract Panel (BluePrint Genetics)
M72	III-3	AD	4	CUSUM	Normal	Not done	Left esotropia 25 prism dioptres	Bilateral congenital cataracts, Esotropia, Amblyopia	Mild to moderate hearing loss, developmental delay, 3–10th %ile height, 25th %ile weight, 2–50th %ile head circumference, micrognathia.	Cataract Panel (BluePrint Genetics)
M73	II-1	AR	11	OD 20/150, OS 20/40	Bilateral macular schisis, vitreous veils, mild pigmentary retinopathy. Right eye developed shallow rhegmatogenous inferior retinal detachment, and was treated by scleral buckle procedure, cryotherapy with gas injection.	Bilateral parafoveal thinning of the outer retina layers. Central splitting between the inner nuclear and the outer plexiform layer.	Small residual cone flicker (6.2 microV in OD, and 7.1 microV in OS) with no measurable responses to ISCE standard seriesd ERG in ffERG	Retinal dystrophy, macular schisis	Hearing loss	–

Family numbers and pedigree IDs (PIDs) correspond to Fig. 1.

*CF* counting fingers, *CUSUM* central unsteady and unmaintained, *ERG* electroretinogram, *ffERG* full field ERG, *mfERG* multifocal ERG, *LP* light perception, *OCT* ocular coherence tomography, *OD* ocular dexter, (right eye), *OS* ocular sinister (left eye).

### Clinical investigations

The age at clinical diagnosis, best corrected visual acuity (BCVA), fundus findings and ocular history, spectral domain ocular coherence tomography (SD-OCT) were reviewed and documented based on the availability. Multifocal electroretinography (mfERG) was recorded with the Espion system (Diagnosys, Lowell MA USA) using DTL electrodes according to ISCEV standards (www.ISCEV.org). Full field ERGs (ffERG) were recorded with the UTAS system (LKC, Gaithersburg, MD USA).

## Results

### Solved families

#### Family C137 — choroidal atrophy (Fig. 1)

The proband of family C137 presented at age 72 with chorioretinal atrophy (Table 1). While the peripheral retina was relatively preserved, bilateral macular depigmentation and moderate vascular attenuation were noted. Visual acuity was counting fingers (CF) in the left eye (Oculus Sinister; OS), and 20/800 in the right eye (Oculus Dexter; OD). Clinical investigation led to molecular investigations for choroideremia. Full sequencing of the coding region of *CHM* revealed no variants, and Western analysis showed normal REP1 protein expression in lymphoblastoid cells (data not shown). WES Results: WES analyses were compared with a list of known retinal genes and revealed a heterozygous variant within *RPE65* (c.1430G > A: p.(D477G)). No other variants of interest were noted.

#### Family M59 — retinitis pigmentosa (Fig. 1)

Two males of Family M59 were diagnosed in early adolescence with retinitis pigmentosa (RP) characterized by night blindness, and peripheral vision loss (Table 1). OCT scan for II-1 showed irregularity of the outer retina with extensive atrophy of the ellipsoid zone and deposits at the level of the Bruch’s membrane-RPE complex (Supplementary Fig. 3B). Panel testing for autosomal recessive RP (Asper Biotech, Tartu Estonia) and X-linked genes (eyeGENE^®^) were negative. WES Results: WES revealed compound heterozygous variations within *BBS1* (c.1169 T > G: p.(M390R) and c.1040del: p.(M347fs)) in both affected males (Table 2). Only the c.1040del variant was noted in the unaffected sister. Follow-up of this family revealed that both brothers suffered from mild learning disabilities, and the youngest brother had postaxial polydactyly on his right foot that was surgically removed. Neither brother was obese or had kidney problems. Considering the new phenotypic data and our genetic result, the diagnosis was altered from nonsyndromic RP to Bardet-Biedl Syndrome (BBS; OMIM 209900).

**Table 2:** Family list of candidate genes determined through WES analysis.

Gene	Annotation	Zygosity in affected(s)	SNP ID	gnomAD Freq	SIFT	PROVEAN	PolyPhen-2	MutationTaster
*Solved Families*								
Family C137								
RPE65	NM_000329.3:c.1430 G > A:p.(Asp477Gly)	Het	rs1571158279	–	Tolerated	Neutral	Benign	Disease causing
Family M53								
ABCA4	NM_000350.3:c.4469 G > A:p.(Cys1490Tyr)	Het	rs61751402	5.90E-05	Not tolerated	Deleterious	Probably damaging	Disease causing
	NC_000001.11(ABCA4):c.4539 + 2028 C > T:p.(Arg1514Leufs*36)	Het	–	–	–	–	–	–
Family M59								
BBS1	NM_024649.5:c.1169 T > G: p.(Met390Arg)	Het	rs113624356	0.0015	Not tolerated	Deleterious	Posssibly damaging	Disease causing
	NM_024649.5:c.1040delT:p.(Met347Argfs*27)	Het	–	–	–	–	–	–
*Partially Solved Families*								
Family M73								
*CDH23*	NM_022124.5:c.767 G > A:p.(Arg256His)	Het	rs371646164	5.40E-04	Tolerated	Neutral	Probably damaging	Polymorphism
	NM_022124.5:c.2263 C > T:p.(His755Tyr)	Homo	rs181255269	0.00139776	Tolerated	Neutral	Probably damaging	Disease causing
	NM_022124.5:c.7415 T > A:p.(Ile2472Asn)	Homo	–	–	Tolerated	Neutral	Benign	Disease causing
*PEX6*	NM_000287.3:c.1802G > A:p.(Arg601Gln)	Het	rs34324426	0.002956	Not tolerated	Deleterious	Probably damaging	Disease causing
*PEX6*	NM_000287.3:c.*437_*445del	Het	rs144286892	0.35810	–	–	–	–
*Putatively Novel Cases*								
Family M54								
LRP1	NM_002332.3:c.650 C > T:p.(Ala217Val)	Het	rs1800127	0.0184	Not tolerated	Neutral	Benign	Polymorpishm
	NM_002332.3:c.9628 G > C:p.(Glu3210Gln)	Het	rs769969579	2.80E-05	Tolerated	Neutral	Probably damaging	Disease causing
Family M70								
LRP1	NM_002332.3:c.2910 G > A:p.(Ser970 = )	Het	rs78054559	1.15E-03	–	–	–	–
	NM_002332.3:c.11930 C > T:p.(Ser3977Leu)	Het	rs142650905	4.67E-04	NA	Deleterious	Probably damaging	Disease causing
Family M72								
STUM	NM_001003665.3:c.62 A > G:p.(Asp21Gly)	Het	Novel	–	Not tolerated	Neutral	Benign	Polymorphism
TOP2A	NM_001067.3:c.2321 A > T: p.(Asn774Ile)	Het	rs61756342	0.001176	Not tolerated	Deleterious	Possibly damaging	Disease causing
UBE2U	NM_152489.1 c.122 A > C:p.(Glu41Ala)	Het	Novel	–	Tolerated	Deleterious	Probably damaging	Disease causing

Only variants in interesting or potential candidate genes are listed (full variant list can be found in Supplementary Table 1 & 2).

#### Family M53 — macular dystrophy (Fig. 1)

The proband of family M53 was initially diagnosed with a cone-dystrophy. At age 12, he had BCVA 20/400 vision in both eyes with reduced colour vision, hyper-reflective flecks in the macula. A ffERG revealed a reduced photopic b-wave. The photopic flicker response was within normal limits as was the scotopic series. The amplitude of the response of the mfERG was reduced, indicating loss of central retinal function. By age 19, a maculopathy was more apparent, with features of degeneration seen with fundus autofluorescence imaging. OCT across the macula showed thinning and atrophy of the ellipsoid zone (Supplementary Fig. 3A). WES Results: WES revealed a single previously known recessive pathogenic variant c.4469 G > A:p.(C1490Y) in *ABCA4* in Stargardt macular dystrophy (OMIM 248200) [9]. This variant was heterozygous in the mother, and not present in the unaffected father. A second variant was found through a larger study of *ABCA4* deep-intronic variants by our collaborators and was noted as c.4539 + 2028 C > T: p.(R1514Lfs*36) [10].

### Partially solved

#### Family M73 — macular schisis and hearing loss (Fig. 1)

The 11-year-old proband of Family M73 presented with bilateral macular schisis and sensorineural hearing loss (SNHL). When diagnosed at age of 11, his BCVA was 20/150 on the right side and 20/40 on the left side. The descriptions for his fundus (Supplementary Fig. 2A) and OCT scan (Supplementary Fig. 3G) are included in Table 1. Chromosomal analysis and testing for known hearing loss genes were negative. ERG revealed only a residual cone flicker response, thus ruling out X-linked juvenile retinoschisis. A blood sample was obtained from the proband and a lymphoblastoid cell line was established via Epstein-Barr transduction. Given the genetic results in this family (described below), a possible peroxisomal biogenesis disorder (Heimler syndrome, OMIM 616617) was investigated. WES Results & cDNA Analysis: WES identified a heterozygous variant in *PEX6*, c.1802G > A: p.(R601Q) previously associated with Heimler Syndrome, a mild peroxisomal biogenesis disorder (OMIM 616617) [11, 12]. No nail or dental abnormalities were noted, and blood pipecolic/plasmalogen levels were normal. cDNA was isolated from a lymphoblastoid cell line, and the coding region of *PEX6* was sequenced; no second coding or splicing variant was noted (data not shown). All variants can be found in Supplementary Table 1. As Falkenberg et al. [13] suggested an allelic expression imbalance can occur in the presence of a common 3’-UTR single-nucleotide polymorphism (SNP, rs144286892), we tested this specific region of the *PEX6* 3’-UTR. Sanger sequencing confirmed the proband is heterozygous for this SNP, which is in agreement with the allelic expression imbalance theory (Supplementary Fig. 1). However, the father (I-1) who passed the c.1802 G > A variant to the proband was also a heterozygote for rs144286892 and had no reported vision or hearing problems. This leaves this case partially solved.

### Putatively novel findings

#### Family M54 and M70 — macular drusen (Fig. 1)

**Fig. 2:**
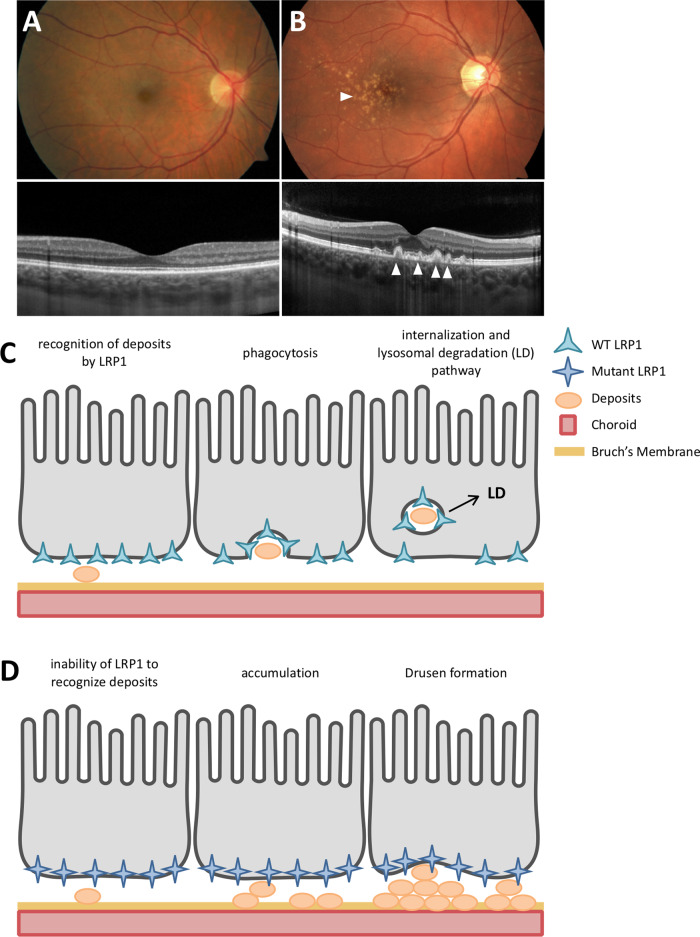
Clinical presentation of M54 proband and LRP1 hypothesis. **A** Normal fundus photo (top) and normal optical coherence tomography (OCT; bottom). **B** Fundus photo (top) of the proband from family M54 illustrating multiple large, confluent drusen in the central macula (white arrow) and nasal retina. OCT (bottom) shows numerous sub-RPE deposits in the macula consistent with drusen (white arrows). (**C**) and (**D**) Hypothesis of LRP1 involvement in MD: as extracellular matter accumulates in Bruch’s membrane, LRP1 endocytoses and destroys this matter. As LRP1 is mutated, this process slows, or is unable to bind particular ligands, leading to accumulated extracellular material, and drusen formation.

The proband of Family M54 was a 44-year-old female who presented with marked bilateral symmetrical macular drusen (Fig. 2B). The retina was otherwise normal, with no signs of degeneration. No other family members were affected, leading to a presumed recessive mode of inheritance. Family M70 presented with one affected female with similar bilateral macular drusen at age 36. OCT scan also showed numerous hyporeflective elevations of the retinal pigment epithelium in the central macula consistent with drusen (Supplementary Fig. 2E). The proband’s mother and sister had no retinal phenotype and a recessive mode of inheritance was considered. The father was unavailable for examination or for a DNA sample. WES Results: WES presumed that the appearance of drusen represented a heritable macular disorder with a relatively higher MAF ( < 0.02) as the trait is not rare in later age groups. Analysis of family M54 revealed compound heterozygous variations in *LRP1* ([c.650 C > T]; [c.9628 G > C], Table 2). Compound heterozygous variants were also found in *CPAMD8* ([c.1030 G > A];[c.5305 C > A]). Compound heterozygous variants within *LRP1* were also noted within the proband of Family M70 ([c.2910 G > A];[c.11930 C > T]). *In silico* analyses can be found in Table 2, and all variants are listed in Supplementary Table 1.

#### Family M72 — congenital cataracts, hearing loss, retinoschisis (Fig. 1)

The affected mother and three affected offspring of Family M72 (sons age 3 and 11, daughter age 7) were referred with dominantly inherited learning disabilities, facial dysmorphism, congenital cataracts, and congenital hearing loss (Table 1). OCT scan from II-1 showed splitting between the inner nuclear and the outer plexiform layer, consistent with retinoschisis (Fig. 4B). WES Results Analysis assumed a dominant mode of inheritance. WES showed no variants in known disease-related genes, and revealed several novel variants of interest, namely, variants in *STUM* c.62 A > G: p.(D21G) and *UBE2U* c.122 A > C: p.(E41A). *In silico* analyses of the *STUM* variant indicated that the variant was likely benign, though analyses predicted the *UBE2U* p.(E41A) variant to be pathogenic (Table 2). See Supplementary Table 1 for all identified variants.

### Unsolved families

#### Family M68 — macular dystrophy with normal fundus (Fig. 1)

The proband of family M68 presented with 20/40 vision in both eyes with −3 dioptres of myopia. A ffERG noted normal cone function and a mild reduction in the pure rod response. A mfERG was reduced in comparison to normal reflecting a retinopathy predominantly affecting the macula but not affecting overall cone function. Gradually the myopia progressed to −7 in both eyes and vision dropped to 20/150 (corrected). The fundus showed mild temporal pallor of the disc with normal SD-OCT imaging. He was labelled clinically as having occult macular dystrophy. WES Analysis: Further analysis indicated rare or novel variants within eight genes (Table 3). We noted heterozygous variants in two genes known to cause congenital stationary night blindness (CSNB): *GRM6* (c.2092 C > G: p.(L698V)) and *TRPM1* (c.3958 G > A: p.(E1320K)). These variants segregated in a ‘digenic’ fashion, and only the *TRPM1* variant was present in the unaffected sister. A heterozygous variant c.1148del in *CNGB3* was also noted though a second variant in *CNGB3* was not discovered through WES.

**Table 3:** WES variant list of unsolved cases from our WES study. This includes all identified variants and *in silico* analyses.

Gene	Annotation	Zygosity in affected	SNP ID	MAF (gnomAD)	SIFT	PROVEAN	Polyphen-2	MutationTaster
*Unsolved Cases*								
Family M68								
*AMER1*	NM_152424.3:c.2175 T > G:p.(Asp725Glu)	Hemi	rs369699027	1.3 × 10-4	Tolerated	Neutral	Benign	Polymorphism
ARAP2	NM_015230.3:c.1393 G > A:p.(Val465Ile)	Het	rs766640340	5.3 × 10-5	Not tolerated	Neutral	Benign	Polymorphism
	NM_015230.3:c.266 C > T:p.(Pro89Leu)	Het	rs35822395	0.008732	Tolerated	Neutral	Benign	Polymorphism
*AVPR2*	NM_000054.4:c.742 C > T:p.(Arg248Cys)	Hemi	rs782516523	1.1 × 10-4	Tolerated	Neutral	Benign	Polymorphism
*CRIPAK*	NM_175918.3:c.52_53insTGCCCATGTGGAGTGCCCGCCTGCTCACACA:p.(Cys18Leufs*400)	Homo	Novel	–	–	–	–	–
	NM_175918.3:c.295_323del29:p.(Cys110Argfs*288)	Homo	Novel	–	–	–	–	–
CSMD2	NM_001281956.1:c.8876 G > A:p.(Gly2959Glu)	Het	rs775668873	1.8 × 10-5	Tolerated	Neutral	Benign	Disease causing
	NM_001281956.1:c.6755 G > A:p.(Arg2252Gln)	Het	rs140769172	0.002683	Not tolerated	Neutral	Possibly damaging	Disease causing
*GRM6*	NM_000843.4:c.2092 C > G:p.(Leu698Val)	Het	rs62638623	0.004705	Tolerated	Neutral	Possibly damaging	Disease causing
DNAH7	NM_018897.2:c.6161 A > G:p.(Tyr2054Cys)	Het	rs62623377	0.01759	Not tolerated	Deleterious	Probably damaging	Disease causing
	NM_018897.2:c.1139 T > G:p.(Met380Arg)	Het	rs144390858	0.009469	Not tolerated	Deleterious	Possibly damaging	Disease causing
*HS6ST2*	NM_001077188.1:c.338 C > T:p.(Ala113Val)	Hemi	rs761589939	5.9 × 10-5	Not tolerated	Neutral	Benign	Polymorphism
OTOG	NM_001277269.1:c.4642 C > T:p.(Leu1548Phe)	Het	rs117380920	0.00801	Tolerated	Neutral	Benign	Polymorphism
*TRPM1*	NM_001252020.2: c.3958 G > A:p.(Glu1320Lys)	Het	Novel variant	–	Tolerated	Neutral	Probably damaging	Disease causing
Family M69								
CC2D2A	NM_001080522.2:c.1691C > T: p.(Thr564Met)	Het	rs201954181	1.3 × 10^−4^	Tolerated	Neutral	Benign	Polymorphism
CROCC	NM_014675.4:c.5237 G > A:p.(Arg1746Gln)	Het	rs139786167	0.004588	Not tolerated	Neutral	Benign	Polymorphism
	NM_014675.4:c.5654 T > C:p.(Val1885Ala)	Het	rs145138931	0.003329	Not tolerated	Neutral	Probably damaging	Disease causing
COL28A1	NM_001037763.2:c.2321 C > G:p.(Thr774Arg)	Het	rs200507350	3.2 × 10-4	Not tolerated	Deleterious	Probably damaging	Disease causing
	NM_001037763.2:c.1318 G > A:p.(Val440Met)	Het	rs1199369584	3.23E-05	Not tolerated	Neutral	Benign	Polymorphism
FLT4	NM_182925.4:c.3908 G > C:p.(Gly1303Ala)	Homo	rs146806202	0.003101	Tolerated	Neutral	Benign	Polymorphism
G3BP2	NM_012297.4:c.970 A > G:p.(Ile324Val)	Homo	rs200985641	8.4 × 10-4	Tolerated	Neutral	Benign	Disease causing
*LCA5*	NM_181714.4:c.401 A > C:p.(Lys134Thr)	Het	rs200395970	3.9 × 10^−5^	Not tolerated	Deleterious	Probably damaging	Disease causing
*MFN2*	NM_014874.4:c.1988G > A:p.(Arg663His)	Het	rs766735605	1.2 × 10^−5^	Not tolerated	Deleterious	Probably damaging	Disease causing
MST1L	NM_001271733.1:c.949 T > C:p.(Trp317Arg)	Het	rs1806514	6.3 × 10-3	Not tolerated	Neutral	Benign	Polymorphism
	NM_001271733.1:c.241 C > G:p.(His81Asp)	Het	rs186420363	Tolerated	Neutral	Possibly damaging	Polymorphism	
MTMR12	NM_001040446.2:c.616 G > A:p.(Asp206Asn)	Homo	rs61748194	0.02048	Not tolerated	Neutral	Possibly damaging	Disease causing
RNF207	NM_207396.2:c.1615C > T:p.(Arg539Cys)	Homo	rs55823245	0.0148	Not tolerated	Deleterious	Probably damaging	Disease causing
*SAMD11*	NM_152486.4:c.222 G > T:p.(Glu74Asp)	Het	Novel	–	Not tolerated	Neutral	Probably damaging	Disease causing
TBC1D9	NM_015130.2:c.1409 G > A:p.(Arg470Gln)	Homo	rs768297413	8.1 × 10-5	Tolerated	Neutral	Benign	Disease causing
UCP1	NM_021833.4:c.778 A > T:p.(Thr260Ser)	Homo	rs776076414	2.4 × 10-5	Tolerated	Neutral	Possibly damaging	Polymorphism
Family M71								
*ALKBH5*	NM_017758.3:c.83 G > A:p.(Arg28Gln)	Het	Novel variant	–	Not tolerated	Neutral	Possibly damaging	Disease causing
*ATG2B*	NM_018036.5:c.410 C > G:p.(Thr137Arg)	Het	Novel variant	–	Not tolerated	Neutral	Probably damanging	Disease causing
*ELAVL2*	NM_004432.3:c.804 C > G:p.(Ile268Met)	Het	Novel variant	–	Tolerated	Neutral	Benign	Disease causing
*ELMOD1*	NM_018712.3:c.286 C > G:p.(Pro96Ala)	Het	Novel variant	–	Tolerated	Deleterious	Benign	Disease causing
*KIF20B*	NM_001284259.1:c.2750 A > G:p.(Gln917Arg)	Het	Novel variant	–	Not tolerated	Neutral	Probably damaging	Polymorphism
*LCORL*	NM_001166139.1:c.146 A > G:p.(His49Arg)	Het	Novel variant	–	Tolerated	Neutral	Benign	Disease causing
*TAAR1*	NM_138327.1:c.685 C > A:p.(Gln229Lys)	Het	Novel variant	–	Tolerated	Neutral	Benign	Disease causing
*TECTA*	NM_005422.2:c.3351 C > G:p.(Asp1117Glu)	Het	Novel variant	–	Tolerated	Deleterious	Benign	Disease causing
*WTIP*	NM_001080436.1:c.49 G > T:p.(Gly17Trp)	Het	Novel variant	–	Not tolerated	Neutral	Possibly damaging	Disease causing

#### Family M69 — macular dystrophy (Fig. 1)

The parents of family M69 were first cousins and an autosomal recessive mode of inheritance was prioritized. The two affected offspring (male 32, female 30) presented with a macular dystrophy. OCT scan from II-2 showed atrophy of the outer retina and absence of Bruch’s membrane in the central fovea (Supplementary Fig. 3D). While the brother provided a DNA sample and was not available for examination, he has been described as significantly near-sighted at a young age requiring the use of corrective lenses. WES Analysis: WES comparisons to retinal disease genes identified heterozygous variations in four genes and further analysis identified variants in seven additional genes (Table 3).

#### Family M71 — cataracts and retinal detachment (Fig. 1)

Family M71 exhibited a highly-penetrant, autosomal dominant condition, which spanned three generations with multiple affected individuals affecting primarily males (Fig. 1). The proband was born with left microphthalmia and retinal detachments were noted shortly after birth. When last examined, he had high myopia with bilateral cortical cataracts, which were removed in 2013 and revealed a previously unrecognized retinopathy (Supplementary Fig. 3F). A diagnosis of Wagner syndrome was considered. WES Analysis: WES analysis was carried out on four members (affected father, two affected brothers, and unaffected mother) and assumed dominant inheritance (Fig. 1). No variations were noted in genes known to cause Wagner syndrome or other retinal detachments (*ATOH7*, *TSPAN12*, *LRP5*, or *NDP*) or in genes known to cause ocular disease. WES identified 72 variations shared between all three affected individuals. Of these, novel variants were noted in nine genes (Table 3). All identified variants in family members of M71 can be found in Supplementary Table 2.

## Discussion

WES provides a cost-effective analysis for clinical investigation in families with IRDs as well as research studies aiming to identify novel retinal disease genes. Our study used WES to study 10 families from Alberta, Canada, chosen from a database of ophthalmology patients (IMM) based on the likelihood of identifying novel genetic associations in retinal and ocular disease. Below we discuss these results in context of our genetic and clinical findings. We also describe potential genetic associations in our unsolved cases that provide insights for other investigators of these blinding conditions.

### Solved cases

#### Choroidal atrophy

Clinical review of this patient indicated a likely diagnosis of choroideremia, however, molecular testing of *CHM*/REP1 precluded this diagnosis. Our results identified a *RPE65* variant (c.1430 G > A: p.(D477G)) previously shown to cause choroidal atrophy [14]. Though *RPE65* variants cause recessive Leber congenital amaurosis, this variant has a unique effect and is the only *RPE65* variant known to cause a choroideremia-like appearance. Functional studies of the p.(D477G) variant have shown that the protein variant facilitates mono- and di-ubiquitination of the RPE65 protein [15], and another showed that c.1430 G > A leads to aberrant RNA splicing [16]. We suggest that cases of choroideremia with negative *CHM* sequencing should be tested for this *RPE65* variant.

#### Bardet-Biedl syndrome

Our analysis identified two variants in *BBS1* (c.1169 T > G: p.(M390R) and c.1040del: p.(M347fs)). The affected males were initially diagnosed as non-syndromic RP but the presence of known *BBS1* variants, as well as additional family information (presence of post-axial polydactyly and learning disabilities), altered our diagnosis to Bardet-Biedl Syndrome, a multi-system ciliopathy. The c.1040del variant was previously reported in a large study of BBS1 [17]. In addition, the p.(M390R) variant has been reported to cause a wide spectrum of phenotypes from non-syndromic RP to severe BBS [18] when inherited in a recessive manner. This suggests that genetic modifiers or mutational burden influences phenotypic presentation, a phenomenon that has been documented to impact presentation in ciliopathies such as BBS [19, 20].

#### Stargardt macular dystrophy

We identified a known pathogenic recessive variant in *ABCA4* (c.4469 G > A: p.(C1490Y)) in the proband of family M53. This prompted a review of clinical data, as well as a search for the second variant, which ultimately identified a deep intronic variant through a collaborator c.4539 + 2028 C > T: p.(R1514Lfs*36) [10]. Combined, this data led us to diagnose Stargardt macular dystrophy, an adolescent-onset maculopathy. This case illustrates the importance of identifying heterozygous variants in WES data for recessive conditions, which may act as ‘guides’ for further analysis.

#### Peroxisomal biogenesis

WES analysis of family M73 identified a single variant in *PEX6*, c.1802G > A: p.(R601Q). This variant has been previously associated with a mild peroxisomal biogenesis disorder called Heimler syndrome [12], characterized by macular schisis, SNHL, and dental/nail abnormalities. Although the proband of this family presented with SNHL and macular schisis, no nail or dental abnormalities were identified and normal pipecolic/plasmalogen were noted in blood. These findings indicate that this is either a mild presentation of a peroxisomal disorder, or that our finding is coincidental. cDNA analysis showed no splicing abnormalities; however, this analysis was carried out in EBV transformed lymphoblast cells and may not contain the appropriate *PEX6* isoform. It is possible that a second variant is controlling expression of the *PEX6* gene through a regulatory sequence, which may be detectable through targeted or NGS.

### Putatively novel findings

#### Heritable macular drusen (Families M54 & M70)

**Fig. 3:**
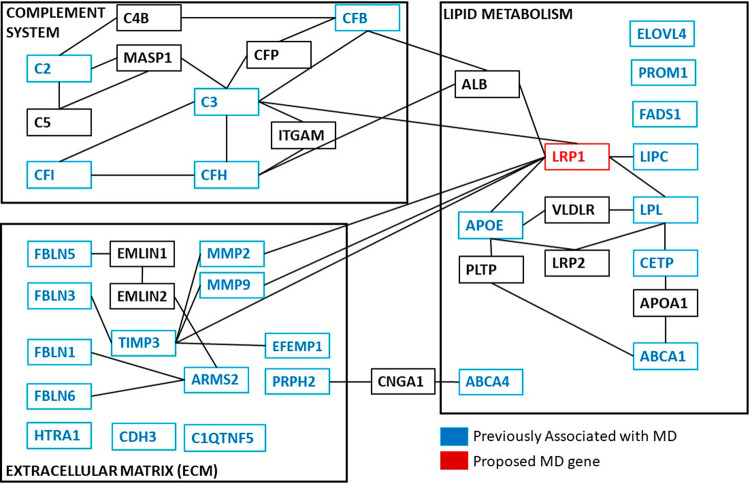
Pathways involved in Macular Degeneration/Dystrophy pathogenesis. The genetics of MD/dystrophy can be broken into three biological processes: (1) ECM homeostasis and remodeling (2) Lipid metabolism (3) Complement system. Proteins implicated in macular dystrophies or MD are highlighted in blue. LRP1 (red) through interaction with known proteins, provides a fascinating bridge between these three processes. Adapted from Kortvely and Ueffing [36].

Drusen are subretinal deposits of lipids and proteins that are a major risk factor for age-related macular degeneration (AMD), though the exact relationship between drusen and photoreceptor death is not clear. We report two pedigrees with early-onset (<40 years of age) bilateral, macular drusen and compound heterozygous variants in *LRP1:* Family M54 with [c.650 C > T];[c.9628 G > C] and M70 with [c.2910 G > A];[c.11930 C > T]. One study showed that the c.650 C > T variant may activate a cryptic microRNA binding site, leading to altered expression of *LRP1*. Although the c.2910 G > A:p.(S970 = ) variant is synonymous, *in silico* splice predictions (SplicePort and Human Splice Finder) for the c.2910 G > A variant predict that this likely creates an exonic splice suppressor and alters an exonic splice enhancer near the variation. *LRP1* is an intriguing candidate gene in the pathology of drusen formation. First, it has direct interaction with many components of drusen and proteins associated with MD, such as amyloid-beta [21], APOE, complement factors, and components of lipid metabolism. Second, LRP1 is expressed in the retinal pigment epithelium (RPE). The RPE provides nutrients to the neural retina, and the basal RPE is the site of drusen formation. Third, LRP1 provides a fascinating link between the three pathologies involved in age-related MD: lipid metabolism, complement pathway, and extracellular matrix homeostasis (Fig. 3). We hypothesize that dysfunction of LRP1 protein leads to accumulation of extracellular material over time due to altered endocytosis kinetics, resulting in drusen. Due to its multiple roles in MD-related pathways and our interesting genetic results, we hypothesize that LRP1 plays a role in drusen formation. Further studies of this protein and its relationship to drusen formation and MD are necessary.

#### Novel syndrome with cataracts, hearing loss, and intellectual delays (Family M72)

**Fig. 4:**
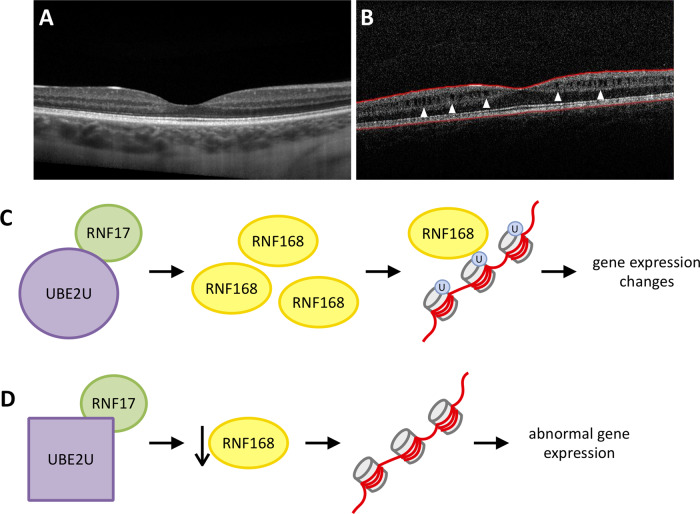
Family M72 Phenotype and UBE2U hypothesis. **A** OCT from an unaffected individual through the macula. **B** OCT scan from II-1 shows splitting between the inner nuclear and the outer plexiform layer, consistent with retinoschisis (white arrows). **C** UBE2U has previously been shown to interact with the RIDDLE syndrome protein RNF168. **D** We hypothesize that the variant in UBE2U identified in Family M72, causes a lack of physical interaction with RNF168 and other interactors, leading to a RIDDLE-like syndrome.

To the best of our knowledge, Family M72 presents a novel dominantly inherited condition. WES identified two novel variants: c.62 A > G: p.(D21G) in *STUM*, and c.122 A > C: p.(E41A) in *UBE2U*. Of interest, UBE2U has been reported to regulate RNF168 [22], an E2-ubiquitin conjugating enzyme that has been associated with the Radiosensitivity Immunodeficiency Dysmorphic features and Learning difficulties (RIDDLE) syndrome (OMIM 611943). Clinical assessment by a medical geneticist (OC) indicated this family shared some systemic dysmorphisms (short stature, small head circumference, low weight, hypertelorism) and behavioral/learning disabilities, similar to RIDDLE syndrome patients. We predict that the variant leads to an abnormal interaction between variant UBE2U and RNF168 and leads to a RIDDLE syndrome-like phenotype in our patients (Fig. 4). The affected mother of this family also developed breast cancer at age 31. The RNF168 system is involved with the repair of DNA damage and has a physical interaction with BRCA1, the most common cause of genetic breast cancer [23]. The diagnosis of breast cancer in our patient may be unrelated but is an interesting observation in the context of the *UBE2U* variant.

### Unsolved families

#### Macular dystrophy with normal fundus (Family M68)

WES analysis yielded no obvious results to pinpoint the genetic etiology of this retinopathy. Of interest was the heterozygous *CNGB3* c.1148del variant, previously associated with achromatopsia, an autosomal recessive cone photoreceptor disease. However, we excluded the likelihood of a second CNGB3 variant as the electrophysiological results make a diagnosis of achromatopsia unlikely; however, the testing revealed a slightly depressed b-wave, indicating a dysfunction between photoreceptors and the interneurons. We identified two heterozygous variants in genes that cause CSNB: *GRM6* (c.2092 C > G: p.(L698V)) and *TRPM* (c.3958 G > A: p.(E1320K)). Van Genderen et al. (2009) suggested that these two proteins directly interact and that TRPM1 is channel-gated by the GRM6 signaling pathway [24]. We postulate that digenic inheritance of these variants is the potential cause of this inherited retinopathy, though further segregation or functional testing is needed to confirm this.

#### Macular dystrophy (Family M69)

The two affected individuals shared variants in many genes not previously associated with IRD (Table 3), however a direct cause is not apparent. The most interesting candidate gene is *CROCC*, which encodes rootletin protein, a core component of the ciliary rootlet [25]. Knockout of rootletin leads to loss of the ciliary rootlet and photoreceptor degeneration in mice [26, 27]. Our reported patient variants may underlie the patient phenotype due to a fragile photoreceptor cilium, which could hinder light detection and phototransduction.

#### Cataracts and retinal detachment (Family M71)

Three affected individuals across two generations presented with retinal detachments. WES revealed no variants in known cataract or retinal detachment-associated genes. We observed novel variations in nine potential candidate genes identified by WES (Table 3). Pathogenicity predictor programs and gene expression data further narrowed this to three genes: *ELAVL2, WTIP*, and *ATG2B*. Embryonic Lethal Abnormal Vision-Like 2 (*ELAVL2*) is a neuron-specific RNA binding protein that regulates transcript expression during neuronal development [28, 29]. *ELAVL2* is expressed early during retinal development, coincident with the differentiation of retinal neurons [30, 31]. Another notable gene is Wilms Tumour Interacting Protein (*WTIP*), which is important for cell–cell and cell–extracellular matrix adhesion in the kidney [32]. There is also evidence that WTIP associates with basal bodies of cilia [33]. *WTIP* expression and function have not been investigated in the eye, but it may play a role in adhesion of the retina to the extracellular matrix and cell–cell adhesion in the lens. Autophagy Related 2B (*ATG2B*) is a key component of autophagosome biogenesis [34, 35]. The ATG complex is comprised of ATG2A and ATG2B, which are functionally redundant — therefore, single loss-of-function variants are unlikely to produce a phenotype. However, whether they have overlapping expression in the human eye is unknown. The functional consequence of the patient’s missense variant in *ATG2B* is unknown; however, impaired autophagosome development could result in accumulation of damaged organelles and abnormal proteins, leading to cellular dysfunction and death.

## Conclusions

Our approach used WES to identify novel genes in IRDs and through this work we have identified two putatively novel associations in retinal disease: *LRP1* in drusen formation and *UBE2U* in a novel syndrome. In our experience, WES can provide a valuable tool into the interrogation of particularly difficult to solve cases. In some instances, it can provide a starting point by identifying a single variant in a recessive condition. In other cases, the genetic information provided by WES can establish or change a clinical diagnosis highlighting the need for multi-disciplinary clinical investigations before genetic studies, as some phenotypic features may be missed by a single specialist. In addition, WES can provide a direction for further interrogations in more difficult cases by identification of potentially novel associations.

## Supplementary information

Supplementary Table 1

Supplementary Table 2

Supplementary Figure 1

Supplementary Figure 2

Supplementary Figure 3
