# Supplementary figures and images for: Whole exome sequencing reveals putatively novel associations in retinopathies and drusen formation
Source: Eur J Hum Genet. 2021 Mar 29;29(8):1171–85. doi: 10.1038/s41431-021-00872-3 (PMC8385108; doi:10.1038/s41431-021-00872-3)

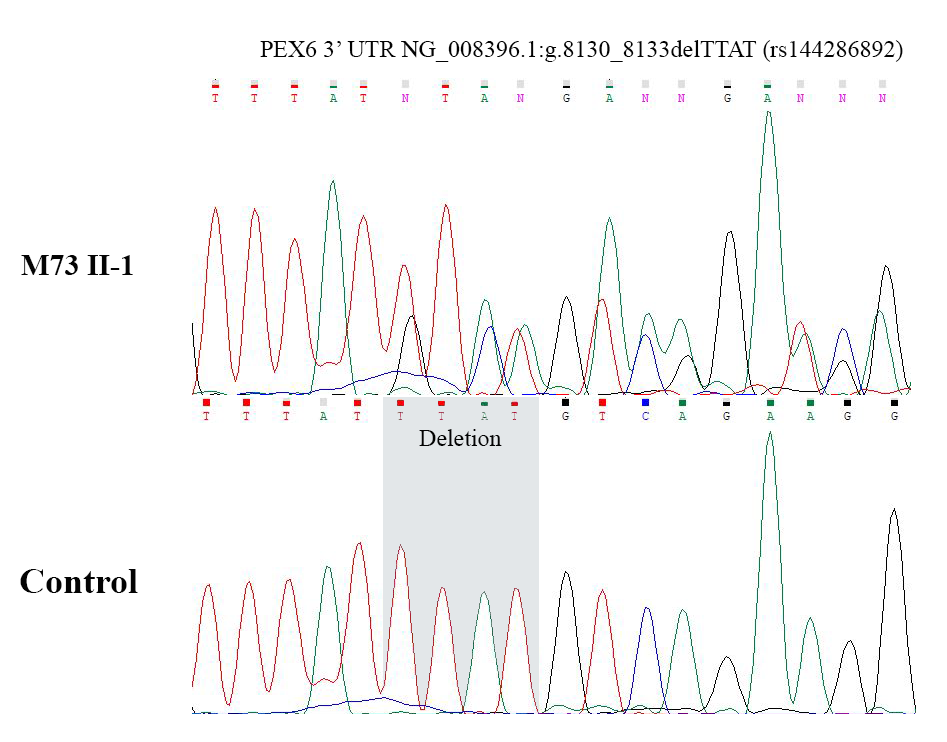

Supplement: Supplementary file 3 — Supplementary Figure 1 [file 41431_2021_872_MOESM3_ESM.tif]

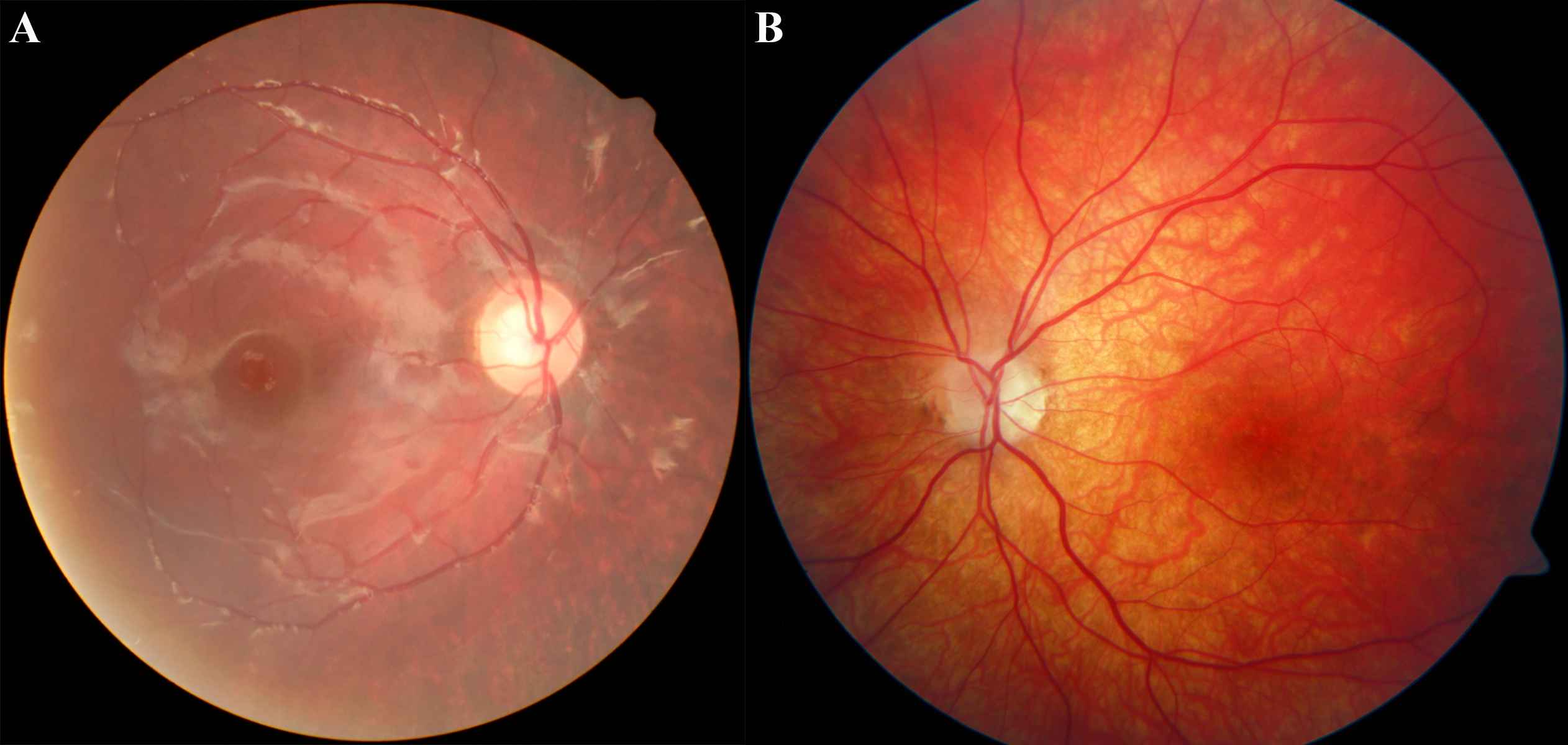

Supplement: Supplementary file 4 — Supplementary Figure 2 [file 41431_2021_872_MOESM4_ESM.tif]

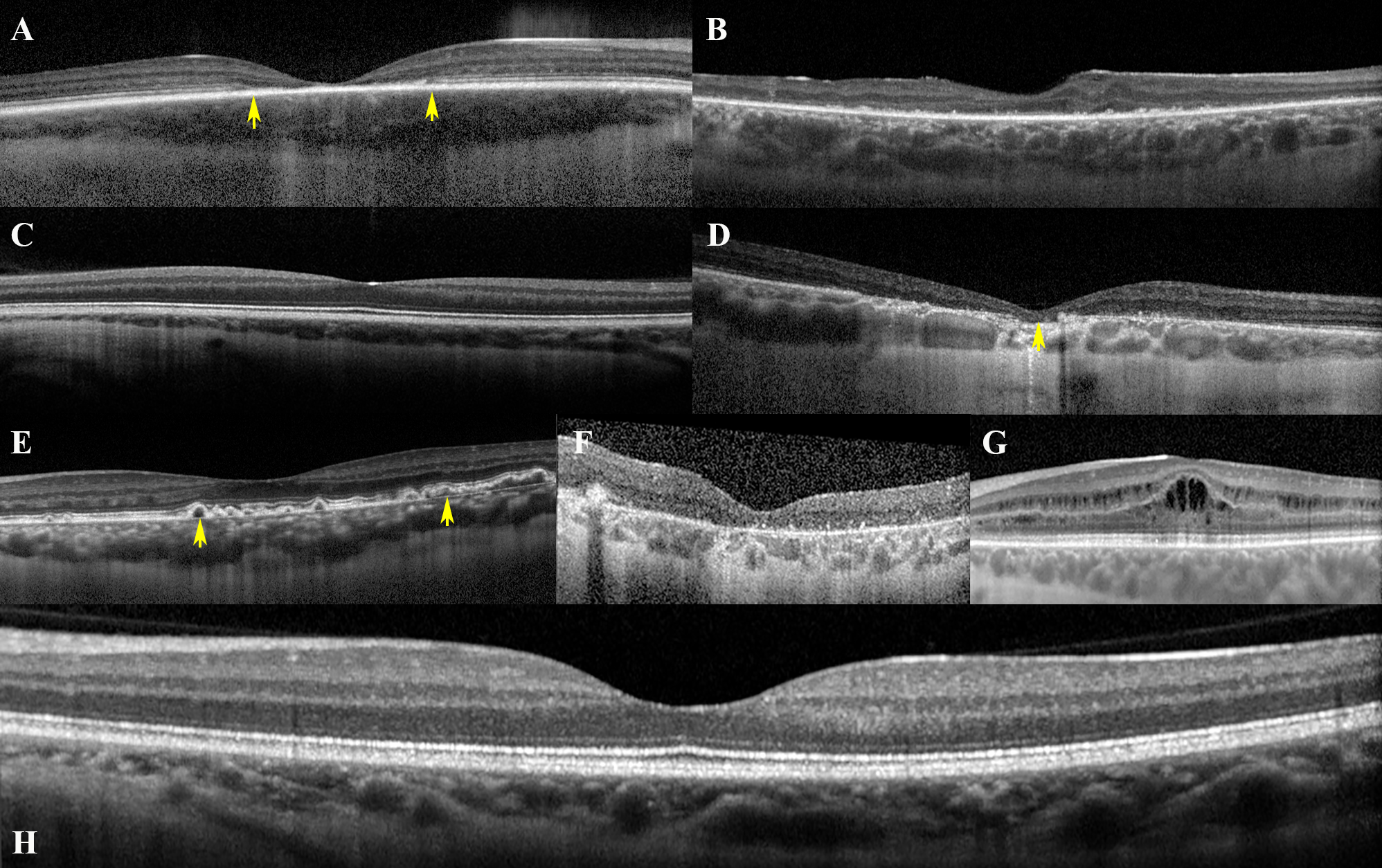

Supplement: Supplementary file 5 — Supplementary Figure 3 [file 41431_2021_872_MOESM5_ESM.tif]
